# Impairments in error processing and their association with ADHD symptoms in individuals born preterm

**DOI:** 10.1371/journal.pone.0214864

**Published:** 2019-04-11

**Authors:** Anna-Sophie Rommel, Sarah-Naomi James, Gráinne McLoughlin, Giorgia Michelini, Tobias Banaschewski, Daniel Brandeis, Philip Asherson, Jonna Kuntsi

**Affiliations:** 1 King’s College London, Social, Genetic and Developmental Psychiatry Centre, Institute of Psychiatry, Psychology and Neuroscience, London, United Kingdom; 2 Icahn School of Medicine at Mount Sinai, Department of Psychiatry, New York, New York, United States of America; 3 Medical Research Council Unit for Lifelong Health and Ageing at University College London, London, United Kingdom; 4 Department of Child and Adolescent Psychiatry and Psychotherapy, Central Institute of Mental Health, Medical Faculty Mannheim/Heidelberg University, Mannheim, Germany; 5 Department of Child and Adolescent Psychiatry and Psychotherapy, Psychiatric Hospital, University of Zurich, Zurich, Switzerland; 6 Center for Integrative Human Physiology, University of Zurich, Zurich, Switzerland; 7 Neuroscience Center Zurich, University of Zurich, Zurich, Switzerland; Universtiyt of Oviedo (Spain), SPAIN

## Abstract

Preterm birth is associated with heightened risk for attention-deficit/hyperactivity disorder (ADHD)-like symptoms and neurocognitive impairments, including impairments in performance monitoring. Here, we investigate the cognitive and neurophysiological processes from a performance-monitoring task in preterm-born adolescents and examine whether these processes in preterm-born adolescents reflect identical neurophysiological impairments to those observed in term-born adolescents with ADHD. We compared 186 preterm-born individuals to 69 term-born individuals with ADHD and 135 term-born controls on cognitive-performance measures and event-related potentials (ERPs) of conflict monitoring (N2) and error processing (ERN, Pe) from a flanker task. Preterm-born adolescents demonstrated reduced N2, ERN and Pe amplitudes, compared to controls, and similar ERN and Pe impairments to term-born adolescents with ADHD. While ADHD symptoms correlated with ERN amplitude at FCz among the preterm-born, ERN amplitude at Fz, N2 and Pe amplitude were not associated with ADHD symptoms. Preterm-born individuals show impairments on neurophysiological indices of conflict monitoring (N2) and error processing (ERN and Pe). Early neurophysiological error processing may be a marker underlying the processes linked to the increased risk for ADHD among preterm-born individuals. Error detection processes are malleable and potential targets for non-pharmacological interventions. Preterm-born individuals are likely to benefit from early interventions.

## Introduction

An estimated 15 million babies worldwide are born preterm (before 37 completed weeks of gestation) every year, with a mean preterm birth rate of 8.6% in the developed world [1]. Although advances in neonatal intensive care have improved survival rates among preterm-born individuals [2], the survivors are at heightened risk of adverse long-term outcomes [3,4] because preterm birth entails biological immaturity for extrauterine life. However, there are no direct measures of degree of maturity. While pregnancy duration and fetal growth are interrelated, a biologic continuum exists, and similar fetal sizes may not indicate similar levels of maturity. As a result, gestational age, which denotes the duration of the pregnancy, is used as a proxy [5]. Preterm birth has been identified as biological insult [6], elevating risk for developing ADHD 1.3 to 5-fold [3,4].

Preterm-born individuals are not only at an increased risk for the behavioral symptoms of ADHD, but also for similar cognitive impairments as observed in individuals with ADHD [3,4], such as impairments in attention and inhibitory control [7–10].

Cognitive-performance data, however, only affords indirect insight into covert processing. Electrical potentials generated by the brain following internal or external events (such as stimuli and responses), known as event-related potentials (ERPs) [11], instead, permit direct examinations of covert brain processes with millisecond temporal resolution [12]. A comparison of the cognitive-neurophysiological profiles of individuals born preterm and individuals with ADHD can, therefore, elucidate if the symptoms and impairments associated with preterm birth are identical to those linked with ADHD or whether they are part of wider-ranging impairments. Accordingly, neurophysiological assessments have the potential to elucidate markers associated with the increased risk for ADHD in preterm-born individuals.

ERP studies in preterm-born individuals in childhood, adolescence and adulthood are scarce. Most ERP research carried out in preterm-born children has focused on auditory ERP measures [13–15]. Auditory ERP studies using the oddball paradigm in very preterm-born children (<32 weeks) demonstrated enhanced N2 amplitude to deviants and interpreted this as indexing impaired attention orienting [13].

Direct comparisons between individuals born preterm and individuals with ADHD are rare but can explicate whether the impairments observed in preterm groups are truly identical to those found in individuals with ADHD or part of wider-ranging impairments. This could aid in identifying biomarkers for the underlying processes linked to the increased risk for ADHD among preterm-born individuals, and effectively in planning targeted interventions. Only one small-scale ERP study (N = 41 across 4 groups, mean age = 8.6 years) investigated attentional processing in preterm-born children of very low birth weight (<34 weeks and <1501g) with and without ADHD, as well as in term-born controls and term-born individuals with ADHD, using a visual oddball paradigm [16]. Compared to term-born controls and preterm-born participants without ADHD, term- and preterm-born children with ADHD demonstrated electrophysiological impairment in motor inhibition (NoGo-N2), [16]. We recently contrasted adolescents and young adults born preterm with term-born controls and term-born individuals with ADHD on cognitive-neurophysiological variables obtained from the cued continuous performance task (CPT-OX). The CPT-OX sensitively measures attentional and inhibitory impairments in individuals with ADHD [17]. On this task, the preterm-born individuals demonstrated electrophysiological impairments in response preparation (CNV), executive response control (Go-P3) and response inhibition (NoGo-P3). While the executive response control (Go-P3) impairments were observed only in the preterm group, impairments in response inhibition (NoGo-P3) and response preparation (CNV) were also found in term-born individuals with ADHD. The NoGo-P3 impairments found in the preterm group were significantly associated with higher ADHD symptoms. These findings suggest that preterm birth may represent a risk factor for ADHD, as well as for additional impairments.

In addition to impairments in attention and inhibition, neurocognitive impairments in ADHD include deficits in performance monitoring [18,19]. The ability to monitor on-going performance comprises conflict monitoring and error detection, and is a crucial prerequisite for adaptively altering behavior and decision-making [20]. Behavioral impairments on performance monitoring tasks, namely higher numbers of errors and increased reaction times during the processing of incongruent stimuli, have also been found in very preterm-born individuals [21,22]. Electrophysiological indices of performance monitoring, namely conflict monitoring (N2) and error processing (error-related negativity (ERN) and error positivity (Pe)) are commonly investigated using flanker tasks, which induce response conflict between relevant and irrelevant information. Previous research on performance monitoring in individuals with ADHD has indicated reduced N2 amplitude (indexing conflict monitoring) [19,23–25], as well as reduced ERN and Pe amplitude (indexing error processing) [19,26] compared to controls. No study to date has investigated the ERP components obtained from performance monitoring tasks in preterm-born individuals.

The objective of the current study is to examine the cognitive and neurophysiological processes from a performance-monitoring task in preterm-born adolescents and young adults. Building on the cognitive-performance and ERP measures, which sensitively captured the performance monitoring impairments in adolescents and young adults with ADHD [19], we further aim to establish whether these cognitive and neurophysiological processes in preterm-born individuals reflect similar neurophysiological impairments to those found in term-born individuals with ADHD, by directly comparing individuals born preterm to term-born individuals with ADHD and term-born controls on an arrow flanker task with low- and high-conflict conditions. We also examine whether impairments in preterm-born individuals are related to their ADHD symptoms using correlations between ADHD symptom scores and ERP components in the preterm group.

## Methods and materials

### Sample

The sample comprised 194 adolescents and young adults born preterm (<37 weeks of gestation), 93 ADHD participants and 166 controls. Exclusion criteria for all groups were IQ<70, general learning difficulties, cerebral palsy or any other medical conditions that affects motor co-ordination including epilepsy, as well as brain disorders and any genetic or medical disorder that might mimic ADHD. In addition, preterm birth was an exclusion criterion in the ADHD and control groups, because this study aimed to establish whether the cognitive impairments associated with preterm birth reflect identical neurophysiological impairments in term-born individuals with ADHD.

The preterm group was recruited from secondary schools in Southeast England. All preterm participants had one full sibling available for ascertainment, and were born before 37 weeks’ gestation. Siblings of preterm-born individuals were included in the preterm group if they were also born preterm (before 37 weeks’ gestation) to maximize the number of participants in the preterm group. Term-born siblings of preterm-born individuals were not included in this analysis. Seven individuals from the preterm sample were excluded because medical birth records could not corroborate preterm status (gestational age (GA) < 37 weeks). One individual was excluded because of IQ<70. While preterm-born individuals were unselected for ADHD status, eight preterm-born individuals met diagnostic criteria for a research diagnosis of ADHD. Since here preterm birth is investigated as a potential risk factor for ADHD, preterm-born individuals who demonstrated high levels of ADHD symptoms were not excluded from the analysis (for an analysis without preterm-born individuals who met a research diagnosis for ADHD, see Appendix A in S1 File).

ADHD and control sibling pairs, who had taken part in our previous research [27], were invited to take part in a follow-up study [19,28]. While ADHD-control differences on cognitive and neurophysiological markers of ADHD persistence and remission have been reported previously [19,28], here the ADHD and control groups are compared to a group of preterm-born individuals. All participants had one full sibling available for ascertainment. Participants with ADHD and their siblings were included in the ADHD group if they had a clinical diagnosis of DSM-IV combined-type ADHD during childhood and met DSM-IV criteria for any ADHD subtype at follow-up. Siblings of individuals with ADHD who did not meet DSM-IV criteria for any ADHD subtype at follow-up were not included in this analysis. The control group was initially recruited from schools in the UK, aiming for an age and sex-match with the ADHD sample. Control individuals and their siblings were included in the control group if they did not meet DSM-IV criteria for any ADHD subtype either in childhood or at follow-up.

Six participants from the ADHD-sibling pair sample were excluded from the group analyses because of missing parent ratings of clinical impairment at follow-up. Therefore, their current ADHD status could not be determined. Two additional participants from the ADHD-sibling pair sample were excluded because of drowsiness during the cognitive task. Two participants with childhood ADHD, who did not meet ADHD symptom criteria but met clinical levels of impairment at follow-up, were excluded to minimize heterogeneity in the ADHD sample. Six control participants were removed from the analyses for meeting DSM-IV ADHD criteria based on the parent-rated Barkley Informant Rating Scale [29]. In addition to these exclusions, which are identical to our previous analyses [19,28], we also excluded six participants from the ADHD-sibling pair sample, who were born preterm, as well as eight individuals from the ADHD-sibling pair sample and 25 participants from the control-sibling pair sample, who provided no information on GA.

The groups demonstrated significant differences with regards to age, IQ, gender distribution, GA and ADHD symptom scores (Table 1). The ADHD group showed significantly higher ADHD symptoms and functional impairment than both the preterm group (t = −16.55, df = 178, p<0.001, d = 2.53; t = −17.23, df = 178, p<0.001, d = 2.94, respectively) and control group (t = 20.06, df = 134, p<0.001, d = 3.74; t = 19.70, df = 134, p<0.001, d = 3.72, respectively). The preterm group further demonstrated significantly higher ADHD symptoms and functional impairment than the control group (t = 4.71, df = 213, p<0.001, d = 0.53; t = 3.83, df = 213, p<0.001, d = 0.45, respectively). While 4% of the preterm group received stimulant treatment, 47% of the ADHD group were treated with stimulant medication at the time of assessment. Prior to assessments, a 48-hour stimulant medication-free period was required. All procedures performed in studies involving human participants were approved by the London-Surrey Borders Research Ethics Committee (09/H0806/58) and the National Research Ethics Service Committee London—Bromley (13/LO/0068). Following procedures approved by the London-Surrey Borders Research Ethics Committee (09/H0806/58) and the National Research Ethics Service Committee London—Bromley (13/LO/0068), written informed consent was obtained from individual participants over the age of 16. Participants under the age of 16 provided informed assent and consent for their participation was obtained from a parent or legal guardian.

**Table 1:** Descriptive statistics. First published in Rommel et al. (2017), ECAP, 26(12):1511–1522.

	ADHD	Preterm	Control	z-statistic	df	p-value
	n = 69	n = 186	n = 135	-	-	-
**GA in weeks (SD)**	39.9 (1.4)	33.0 (3.0)	39.9 (1.3)	-23.0	253	<0.001
**GA range in weeks**	37–42	24–36	37–43	-	-	-
**IQ (SD)**	97.7 (13.8)	104.7 (12.3)	110.4 (12.2)	-3.2	253	0.002
**Age (SD)**	18.5 (3.0)	14.9 (1.9)	17.8 (2.1)	-12.0	253	<0.001
**Age range**	12.7–25.9	11.0–20.0	11.9–21.6	-	-	-
**Males %**	88.4	54.3	75.6	4.6	253	<0.001
**Conners’ parent rated ADHD symptom score (SD)**	35.8 (10.6)	11.2 (9.4)	7.0 (5.6)	1.97	253	0.050
**BFIS score (SD)**	16.4 (5.4)	3.7 (4.1)	2.1 (2.5)	-2.23	253	0.027

BFIS = Barkley Functional Impairment Scale

### Measures

#### The Diagnostic Interview for ADHD in adults (DIVA)

The DIVA [30] is a semi-structured interview, which comprises the 18 items making up the DSM-IV symptom criteria for ADHD. The DIVA assesses both childhood and adult ADHD symptoms and impairment.

#### The Barkley Functional Impairment Scale (BFIS)

The BFIS [29] evaluates the levels of functional impairments often linked to ADHD symptoms in five different areas of life (work/education; family/relationship; social interaction; management of daily responsibilities; and leisure activities) on a 10-item scale.

For consistency, parental ADHD symptom ratings on the DIVA and BFIS were used to evaluate ADHD for all participants in the preterm and ADHD groups. Parents were asked to judge their children’s ADHD symptoms and impairments off medication, if participants typically took stimulant medication. Participants received a research diagnosis of ADHD if they met six or more inattention or hyperactivity-impulsivity symptoms on the DIVA and if they scored positively twice or more in at least two areas of impairment on the BFIS. For all participants in the control group, for consistency, parental ADHD symptom ratings on the BFIS were employed to assess ADHD. If control participants scored positively twice or more in at least two areas of impairment on the BFIS, they were excluded from the analysis.

#### IQ

To derive estimates of IQ, we administered the block design and vocabulary subtests of the Wechsler Abbreviated Scale of Intelligence (WASI-I) [31] to all participants. For digit span forward (DSF) and backward (DSB) measures, we conducted the digit span subtest from the Wechsler Intelligence Scale for Children (WISC-III) [32] with participants aged under 16 and the Wechsler Adult Intelligence Scale (WAIS-III) [33] with participants aged 16 or over. Digit span forward assesses short-term verbal memory, whereas digit span backward measures working memory.

#### Flanker task

The task was an adaptation of the Eriksen Flanker paradigm [34] designed to increase cognitive load as used in previous studies [19,25]. In each trial, a central black fixation mark was replaced by a target arrow (a black 18mm equilateral triangle). Participants had to indicate whether this arrow pointed towards the left or right by pressing corresponding response buttons with their left or right index fingers. Two flanker arrows identical in shape and size to the target appeared 22mm above and below the center of the target arrow 100ms prior to each target arrow. Both flankers either pointed in the same (congruent) or opposite (incongruent) direction to the target. Conflict monitoring is maximal during the incongruent condition. When the target appeared, both target and flankers remained on the screen for a further 150ms, with a new trial being presented every 1650ms. Two hundred congruent and 200 incongruent trials were arranged in 10 blocks of 40 trials over 13 minutes. We calculated cognitive-performance measures of mean reaction time (MRT), reaction time variability (standard deviation of RTs) and number of errors (left-right errors occurring when participants chose the wrong left or right response) separately for congruent and incongruent conditions, consistent with our previous study [19].

#### Electrophysiological recording and processing

The EEG was recorded from a 62-channel DC-coupled recording system (extended 10–20 montage), using a 500Hz sampling-rate, impedances under 10kΩ, and FCz as the recording reference. The electro-oculograms (EOGs) were recorded from electrodes above and below the left eye and at the outer canthi. EEG data were analyzed using Brain Vision Analyzer 2.0 (Brain Products, Germany). Raw EEG recordings were down-sampled to 256Hz, re-referenced to the average of all electrodes, and filtered using Butterworth band-pass filters (0.1-30Hz, 24dB/oct). All trials were visually inspected for electrical artifacts or obvious movement, and sections of data containing artifacts were removed manually. Ocular artifacts were identified using the infomax Independent Component Analysis (ICA) algorithm [35]. Sections of data containing artefacts exceeding ± 100μV or with a voltage step greater than 50μV were automatically rejected. Baseline correction was applied using the -300 to -100ms pre-target (-200 to 0ms pre-flanker) interval.

**Fig 1:**
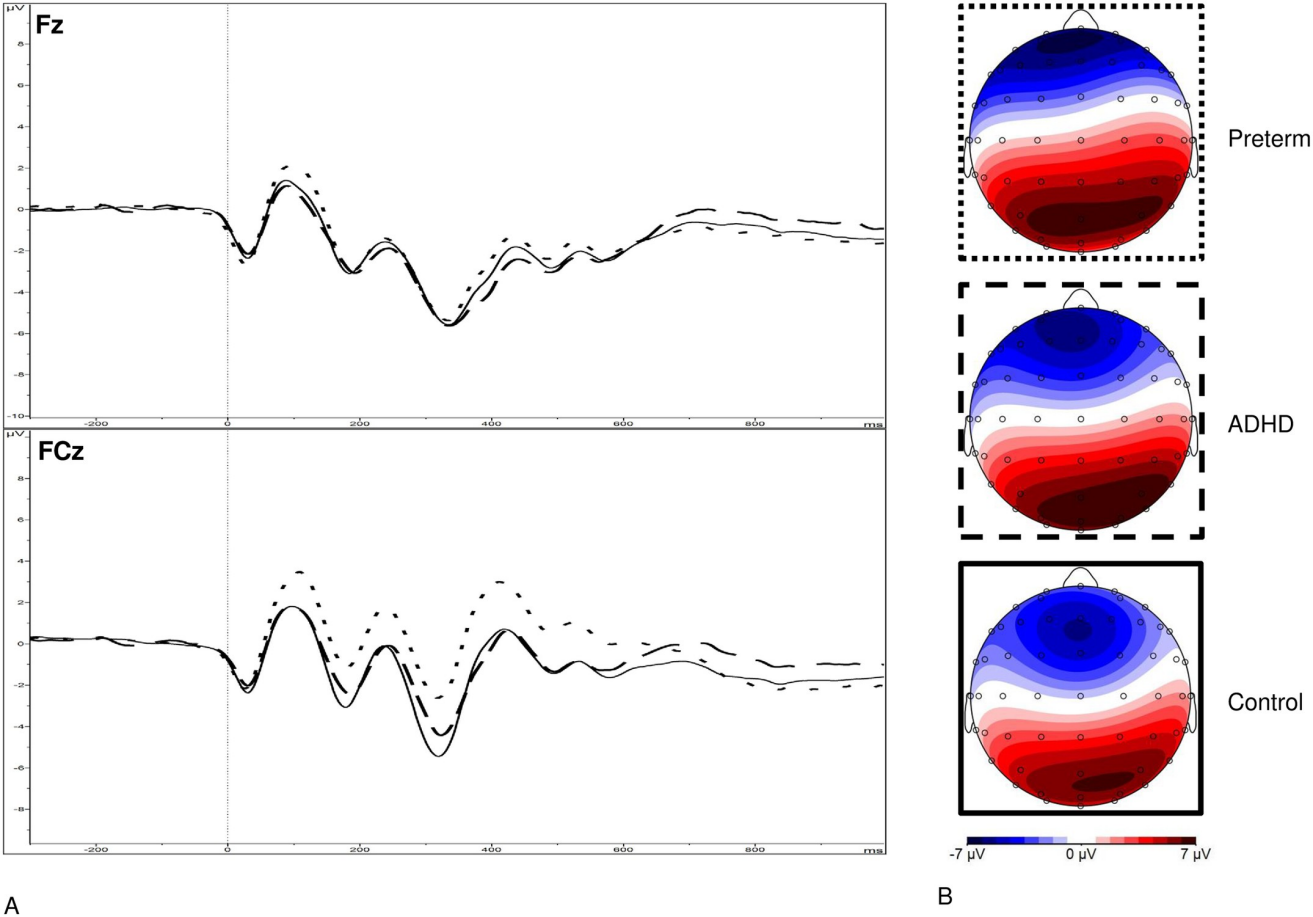
(**A**) Grand average stimulus-locked event-related potentials (ERPs) of the N2 at the Fz and FCz electrodes between 250 and 450 ms after incongruent stimuli where a correct response was made for the preterm group (represented by dotted lines), the ADHD group (attention-deficit/hyperactivity disorder represented by dashed lines) and the control group (shown in solid lines), and (**B**) topographic maps.

**Fig 2:**
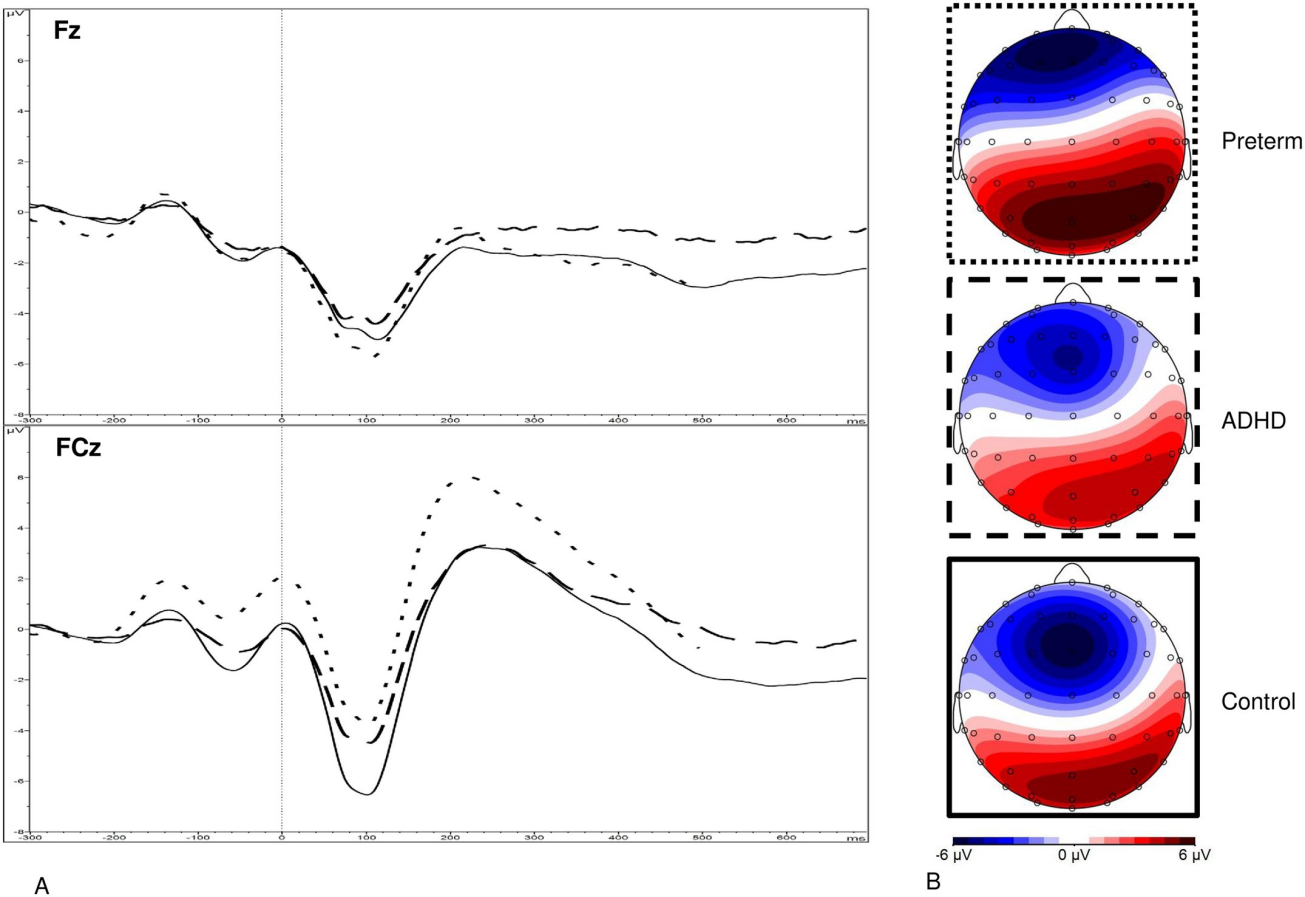
(**A**) Grand average response-locked event-related potentials (ERPs) of the error-related negativity (ERN) at the Fz and FCz electrodes between 0 and 150 ms after an erroneous response on the incongruent trials for the preterm group (represented by dotted lines), the ADHD group (attention-deficit/hyperactivity disorder represented by dashed lines) and the control group (shown in solid lines), and (**B**) topographic maps for each group.

**Fig 3:**
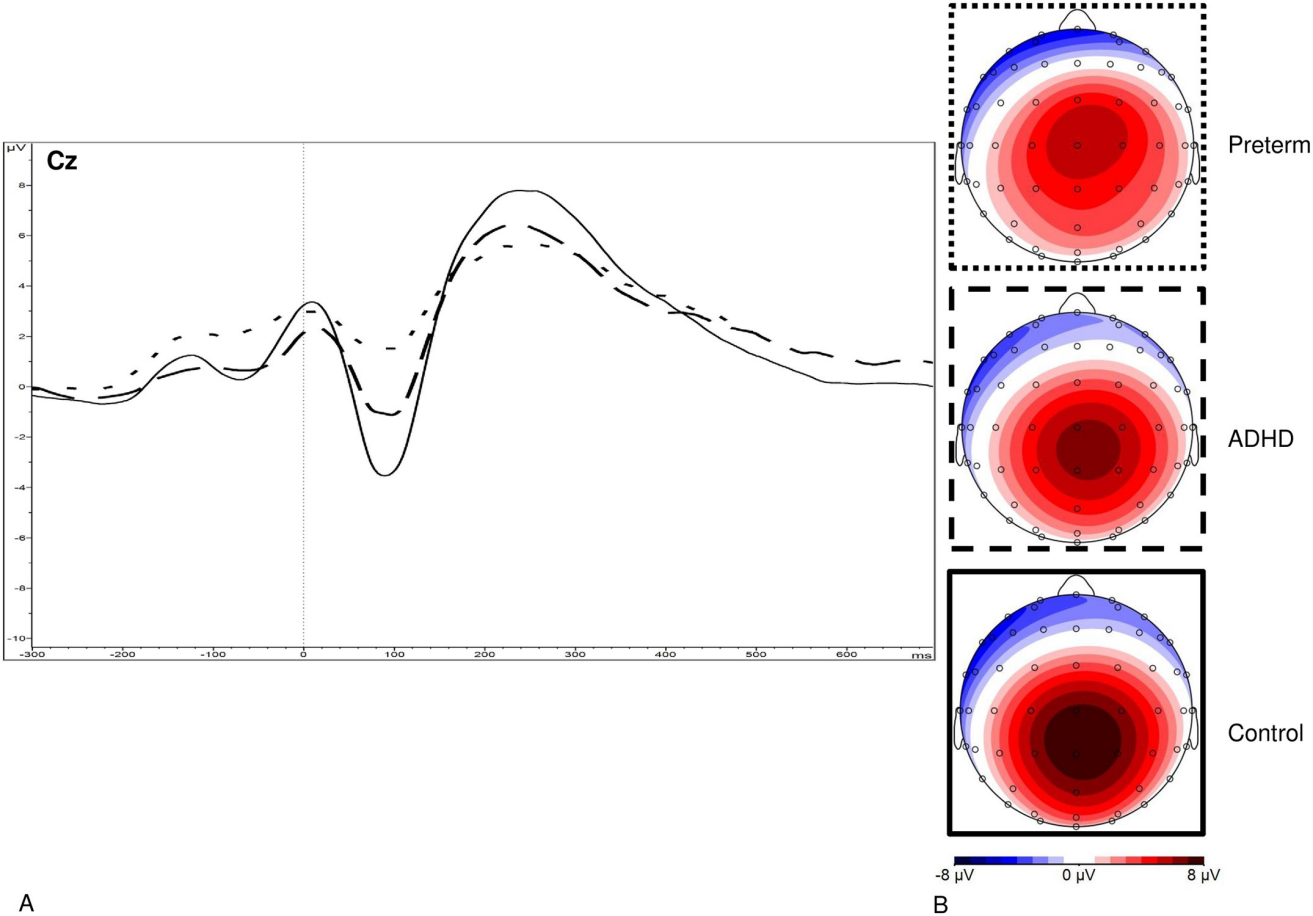
(**A**) Grand average response-locked event-related potentials (ERPs) of the error-related positivity (Pe) at the Cz electrode between 150 and 450 ms for the preterm group (represented by dotted lines), the ADHD group (attention-deficit/hyperactivity disorder represented by dashed lines) and the control group (shown in solid lines), and (**B**) topographic maps for each group.

Data were segmented based on (1) stimulus-locked trials where a correct response was made and (2) response-locked (error-related) trials where an incorrect response was made. Individual averages were created based on each condition, requiring ≥20 clean segments for each participant. After averaging, the electrodes and latency windows for ERP analyses were selected based on previous studies [19,23,25], topographic maps and the grand averages (Figs 1–3). The N2 was measured as maximum negative peak at Fz and FCz between 250-450ms after target onset. The ERN was measured at Fz and FCz between 0-150ms, using a maximal amplitude approach. The Pe was measured as maximum positive peak at Cz between 150-450ms after an erroneous response on incongruent trials.

Two preterm-born participants (1.1%) were excluded from the ERP analysis of the N2 and 14 preterm-born participants (7.5%) were excluded from the ERP analysis of the ERN/Pe due to having fewer than 20 artifact-free segments available for analysis. Two participants with ADHD (2.9%) were excluded from the ERP analysis of the N2 and seven participants with ADHD (10.1%) were excluded from the ERP analysis of the ERN/Pe due to having fewer than 20 artifact-free segments available for analysis. Fourteen control participants (10.4%) were excluded from the ERP analysis of the ERN/Pe due to having fewer than 20 artifact-free segments available for analysis. These exclusion ratios across groups reflect similar ratios reported in previous studies using this paradigm [23,25].

#### Statistical analysis

Data were analyzed using random intercept models in Stata, to control for non-independence of the data (i.e. data coming from siblings of one family), using the ‘robust cluster’ method to estimate standard errors [36]. We ran different regression models for each behavioral performance measure and ERP component (independent variables) with dummy variables to identify which measures showed an overall effect of group (dependent variable; preterm vs. ADHD vs. control), with controls as the reference group. Congruency was included as a covariate in the cognitive-performance measure and N2 models, recording site was included as a covariate in the N2 and ERN models, and interactions were examined. Regression-based corrections for age were applied to raw scores and residual scores were analyzed. Gender was included as a covariate in all analyses. Results are presented both with and without IQ as a covariate to empirically examine the effects of IQ on ERP components. Correlations were also run to examine the associations between impaired ERP measures and DIVA ADHD symptom scores in the preterm group. Effect sizes (Cohen’s d), which were calculated using the difference in the means divided by the pooled standard deviation [37], are reported.

## Results

**Fig 4:**
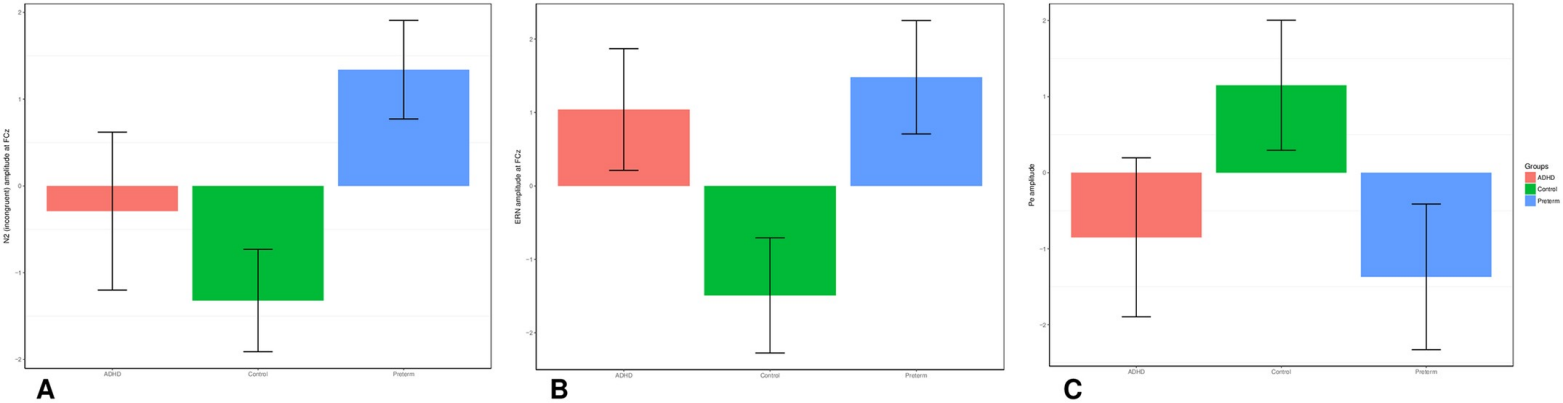
Box plots of the mean amplitude of (A) N2 at FCz, (B) ERN at FCz and (C) Pe. The preterm group is represented in blue, the ADHD group in red and the control group in green. Error bars represent 95% confidence intervals.

The random intercept model yielded a significant main effect of group for MRT (z = -4.16, p<0.001), RTV (z = -3.21, p<0.001), DSB (z = -3.71, p<0.001), N2 (z = 5.35, p<0.001), Pe (z = -2.05, p = 0.041) and ERN (z = 2.90, p = 0.004) amplitude (Figs 1–4). No significant main effect of group emerged for DSF (z = 0.41, 0.682), or errors (z = 0.88, p = 0.377).

Significant group-by-electrode site interactions emerged for N2 (z = 5.03, p<0.001) and ERN (z = 5.55, p<0.001). No significant group-by-congruency interaction emerged for N2 (z = -1.68, p = 0.093), MRT (z = 1.39, p = 0.165), RTV (z = 0.25, p = 0.802) or errors (z = 0.16, p = 0.870). No significant group-by-congruency-by-electrode site interaction emerged for N2 amplitude (z = 0.86, p = 0.390).

Post-hoc analyses found that the preterm group had significantly reduced N2 and ERN amplitude at FCz, as well as reduced DSB and Pe amplitude compared to the control group, with medium-to-large effect sizes (Table 2). Compared to the ADHD group, the preterm groups had significantly decreased incongruent MRT and RTV, as well as reduced N2 amplitude at FCz, medium-to-large effect sizes (Table 2). The preterm group did not differ from the ADHD group on ERN and Pe amplitude (Table 2). The ADHD and control groups significantly differed on DSB, MRT, RTV, N2 amplitude at FCz, ERN amplitude at FCz and Pe amplitude [19].

**Table 2:** Cognitive and ERP measures from the flanker task: means, standard deviations (SD) and effect sizes (Cohen’s d) for the preterm, ADHD and control groups.

		Preterm		ADHD		Control		Group comparison						
		Mean	SD	Mean	SD	Mean	SD							
								p	Preterm vs ADHD		Preterm vs Control		ADHD vs Control	
									p	d	p	d	p	d
**DSF**		0.35 (10.2)	2.1 (2.1)	-0.59 (9.6)	2.0 (2.0)	0.34 (10.5)	2.1 (2.1)	0.682	-	-	-	-	-	-
**DSB**		-0.26 (6.3)	2.1 (2.1)	-0.70 (6.4)	2.2 (2.2)	0.93 (7.9)	2.5 (2.5)	<0.001	0.309	0.21	<0.001	*0*.*50*	<0.001	*0*.*67*
**MRT**	**congruent**	7.1 (346.6)	44.1 (44.8)	39.9 (355.6)	64.1 (64.2)	14.1 (334.7)	32.6 (32.5)	0.063	-	-	-	-	-	-
	**incongruent**	12.2 (445.7)	48.5 (48.1)	44.0 (448.3)	57.1 (57.8)	22.1 (432.4)	40.3 (40.3)	0.011	<0.001	*0*.*62*	0.086	0.23	0.004	0.47
**RTV**		-3.1 (89.2)	42.2 (42.3)	36.3 (116.0)	72.2 (73.5)	-5.4 (75.7)	21.8 (21.8)	<0.001	<0.001	*0*.*75*	0.957	0.06	<0.001	**0.98**
**Errors**		-1.2 (28.9)	15.3 (26.8)	5.8 (34.2)	18.3 (30.2)	-2.4 (26.2)	13.3 (26.2)	0.377	-	-	-	-	-	-
**N2**	**at Fz**	0.50 (-6.2)	4.0 (4.2)	-0.53 (-6.1)	3.0 (3.4)	-0.04 (-5.8)	2.7 (3.0)	0.022	0.053	0.29	0.227	0.17	0.275	0.18
	**at FCz**	1.1 (-3.6)	3.6 (3.7)	-0.4 (-4.6)	3.5 (3.8)	-0.99 (-5.3)	3.2 (3.6)	<0.001	0.006	0.41	<0.001	0.72	0.041	0.30
	**congruent**	0.9 (-5.7)	4.0 (4.3)	-0.4 (-6.2)	3.5 (3.7)	-0.7 (-6.7)	3.3 (3.3)	<0.001	0.002	0.35	<0.001	0.44	0.937	0.03
	**incongruent**	0.6 (-4.1)	3.6 (3.8)	-0.5 (-4.5)	3.1 (3.4)	-0.3 (-4.4)	2.8 (2.9)	<0.001	0.003	0.33	0.015	0.30	0.417	0.06
**ERN**	**at Fz**	0.20 (-7.6)	5.2 (5.2)	1.05 (-5.7)	2.6 (2.4)	0.46 (-6.5)	3.4 (3.4)	0.571	-	-	-	-	-	-
	**at FCz**	1.48 (-5.0)	5.0 (5.0)	1.04 (-6.2)	3.3 (3.2)	-1.49 (-8.6)	4.4 (4.3)	<0.001	0.473	0.09	<0.001	*0*.*62*	<0.001	*0*.*62*
**Pe**		-1.37 (8.0)	6.2 (6.4)	-0.85 (7.6)	4.2 (3.4)	1.15 (10.4)	4.8 (4.9)	<0.001	0.698	0.08	<0.001	*0*.*54*	<0.001	*0*.*60*

Values represent regression-based corrections for age. Raw scores are represented in parentheses. Moderate effect sizes in italics; Large effect sizes in bold; ADHD = attention-deficit/hyperactivity disorder; DSF = digit span forward; DSB = digit span backward; MRT = mean reaction time in ms; RTV = reaction time variability in ms; cong = congruent, incong = incongruent

Among those born preterm, DIVA ADHD symptoms correlated with ERN amplitude at FCz (r = 0.18, p = 0.031), but not with ERN amplitude at Fz (r = 0.10, p = 0.256), Pe amplitude (r = -0.04, p = 0.629), N2 amplitude at Fz (r = 0.14, p = 0.119) or N2 amplitude at FCz (r = 0.02, p = 0.839).

Controlling for IQ, group effects on MRT in the congruent condition became significant (Appendix B in S1 File). Differences between preterm-born individuals and term-born controls became significant for MRT in both conditions. Differences between preterm-born individuals and term-born individuals with ADHD became non-significant for congruent errors (Appendix B in S1 File). Differences between term-born individuals with ADHD and term-born controls became non-significant for incongruent MRT, errors in both conditions and N2 amplitude at FCz [19]. Results for other variables remained unchanged.

Excluding the eight preterm-born individuals meeting criteria for a DSM-IV research diagnosis of ADHD (Appendix A in S1 File), excluding all female participants (Appendix C in S1 File) and matching the groups on age (Appendix D in S1 File) did not change the overall pattern of results.

## Discussion

In this first large-scale comparison of neurophysiological performance monitoring in preterm-born individuals, term-born individuals with ADHD and term-born controls, we demonstrated that preterm-born individuals were impaired on the ERP indices of performance monitoring, including conflict monitoring (N2) and neurophysiological error processing (ERN and Pe). The early (ERN) and late (Pe) error detection impairments observed in the preterm-born individuals were similar to those found in term-born individuals with ADHD. While the impairments in early error processing (ERN) were significantly associated with ADHD symptoms, such that smaller ERN amplitude at FCz was linked to higher ADHD symptom severity, Pe amplitude did not correlate with ADHD symptoms in the preterm group. Early electrophysiological error processing may, therefore, be a putative marker for the underlying processes linked to the increased risk for ADHD among those born preterm.

Reduced N2 amplitude at FCz, indexing conflict-monitoring impairments, was observed in the preterm-born individuals. Previous research had found increased NoGo-N2 amplitude in children born very preterm (<32 weeks) using an auditory oddball paradigm [13]. The discrepancies in the direction of the impairment may be explained by the differences in age, recording site, task and modality investigated. Nevertheless, these findings and our results demonstrate abnormalities in N2 amplitude in preterm-born individuals. Future research is required to further establish N2 impairments in preterm-born individuals and to elucidate these discrepancies. While individuals with ADHD also demonstrated reduced N2 amplitude [19], in line with previous research [23–25], the N2 impairment seen in the preterm group was larger, with the ADHD group lying midway between the preterm and control groups. Moreover, N2 amplitude in the preterm group was not associated with DIVA ADHD symptoms. The reduced N2 component in the preterm group, therefore, does not seem to be linked to the neurophysiological risk for ADHD. Instead, the reduced N2 amplitude in the preterm group could be linked to other neurological or psychiatric morbidities, as well as other risk factors. Previous research has shown, for instance, that individuals with bipolar disorder also exhibit reduced N2 amplitude [38].

Preterm-born individuals further demonstrated impairments in Pe amplitude, compared to term-born controls. While the functional importance of the Pe is still unclear, the Pe has frequently been linked with conscious awareness of having committed an error [39]. In line with this interpretation, the Pe is thought to capture affective responses to making an error, awareness of having committed an error, or processing associated with response adaptation strategies following a mistake [40]. In addition, the amplitude of the Pe has been linked to academic achievement [41] and motivational processes [42] in young children. The Pe impairment in preterm-born individuals did not differ significantly from the impairment seen in term-born individuals with ADHD, who also demonstrated significantly reduced Pe amplitude [19]. However, in the preterm group Pe amplitude did not correlate significantly with DIVA ADHD symptoms. Similarly, to reduced N2 amplitude, reduced Pe amplitude in the preterm group may potentially be related to other neurological or psychiatric disorders. Depression, for example, has previously been linked to reduced Pe amplitude [43,44]. Yet, further research is needed to establish neurophysiological markers underlying the increased risk for other psychiatric disorders following preterm birth.

We further identified impaired early error processing, indexed by reduced ERN amplitude at FCz, in the preterm group. This impairment is similar to that in individuals with ADHD who also demonstrated reduced ERN amplitude compared to controls [19]. The significant correlation between ERN amplitude at FCz and DIVA ADHD symptoms in the preterm group may suggest that greater ERN amplitude is linked to greater ADHD severity. This finding is in line with previous research, which demonstrated behavioral impairments in very preterm-born individuals, such as greater number of errors and increased reaction time, on performance monitoring tasks [21,22]. Impaired early error processing (ERN) may, thus, be a marker of neurophysiological risk for ADHD in preterm-born individuals. One important role of our cognitive system is the ability to monitor and evaluate the outcomes and consequences of behavior and to adapt subsequent behavior accordingly in order to realize goals. To accomplish this, we rely on some form of feedback on our actions. This feedback system is guided by the detection and processing of our errors. It is conceivable that having difficulties with this feedback system (as indexed by neurophysiological impairments in early error detection) and, therefore, with improving performance and with a lack of awareness of behaviors, emotions, and cognitions, may put preterm-born individuals at risk of developing the behavioral and cognitive profile of ADHD. Such difficulties may also manifest during learning or classroom activities, putting individuals at a further cognitive disadvantage. The potential role of early error processing (ERN) as a marker for the underlying processes linked to the increased risk for ADHD among those born preterm should be investigated using longitudinal studies.

We repeated the analyses with IQ as a covariate to examine any potential contribution from IQ to the results. When removing the effects of IQ, differences on MRT and RTV between the groups became non-significant, suggesting that group differences on this measure may reflect impairments related to IQ. These findings may also explain the discrepancy between this study and previous studies [21,22], which found significant cognitive-performance differences between preterm and control groups. Moreover, differences between preterm-born individuals and term-born individuals with ADHD became significant for DSF when controlling for IQ. IQ may, thus, modulate some differences between the groups on cognitive-performance measures. Yet, most results did not change when controlling for IQ, suggesting that, overall, the contribution from IQ to the neurocognitive impairments in preterm-born individuals is limited.

One limitation of this analysis is that we cannot completely rule out the possibility of background factors accounting for our ERP findings, because we were not able to investigate risk factors for preterm birth (e.g., malnutrition, poverty). Yet, population-based quasi-experimental designs found that the link between psychiatric morbidity and preterm birth is largely independent of shared familial confounds and measured covariates, in line with a causal relationship [4]. Nevertheless, risk factors for preterm birth need to be taken into account when investigating cognitive-neurophysiological measures in preterm-born individuals in the future. In addition, other traits and symptoms (e.g., anxiety, depression) may influence performance monitoring. Longitudinal research and additional moderators are needed to further investigate a causal pathway from preterm birth to ADHD. Lastly, the DIVA is a diagnostic interview for adult ADHD but, for consistency, in this study was employed across ages. The DIVA is based on DSM criteria for ADHD. It assesses the presence of current as well as childhood ADHD symptoms in adulthood, and the chronicity of these symptoms. Thus, adaptation for the use in younger participants is practicable.

## Conclusion

This study provides evidence for impairments in performance monitoring, including conflict monitoring (N2) and neurophysiological error processing (ERN and Pe) in preterm-born individuals. While the conflict monitoring impairments in the preterm group do not seem to be identical to those in individuals with ADHD, early neurophysiological error processing may be a biomarker linked to the increased risk for ADHD. Since previous studies have demonstrated that error detection processes are malleable [19] and also potential targets for non-pharmacological interventions [45,46], preterm-born individuals are likely to benefit from early detection of these impairments and timely interventions.

## Supporting information

S1 File Additional analyses. Appendix A–Excluding preterm-born individuals with a research diagnosis of ADHD from the analysis. Appendix B–Analysis controlling for IQ. Appendix C–Analysis of the males only. Appendix D–Analysis of the age-matched subsample.
(DOCX)

S1 Dataset ERN data file. (CVS)
