# Supplementary material for: Impairments in error processing and their association with ADHD symptoms in individuals born preterm
Source: PLoS One. 2019 Apr 11;14(4):e0214864. doi: 10.1371/journal.pone.0214864 (PMC6459538; doi:10.1371/journal.pone.0214864)
Supplement: S1 File — Appendix A–Excluding preterm-born individuals with a research diagnosis of ADHD from the analysis. Appendix B–Analysis controlling for IQ. Appendix C–Analysis of the males only. Appendix D–Analysis of the age-matched subsample. (DOCX) [file pone.0214864.s001.docx]

**Appendix A**

While preterm-born individuals were unselected for ADHD status, eight preterm-born individuals met diagnostic criteria for a research diagnosis of ADHD on the Diagnostic Interview for ADHD in adults. For the following analyses, these individuals were excluded.

The random intercept model yielded a significant main effect of group for MRT (z=-4.62, p<0.001), RTV (z=-3.57, p<0.001), DSF (z=3.87, p<0.001), DSB (z=-2.16, p=0.031), N2 (z=5.02, p<0.001), Pe (z=-3.91, p=0.001) and ERN (z=3.17, p=0.002) amplitude, but not for errors (z=1.10, p=0.273).

Significant group-by-recording site interactions emerged for N2 (z=4.98, p<0.001) and ERN (z=5.60, p<0.001) amplitude. A significant group-by-congruency interaction emerged for N2 (z=-2.21, p=0.027), but not for MRT (z=1.51, p=0.130), RTV (z=0.01, p=0.994) or errors (z=-0.11, p=0.911). No significant group-by-congruency-by-electrode site interaction emerged for N2 amplitude (z=-0.79, p=0.428).

Post-hoc analyses revealed that the preterm group had significantly reduced N2 amplitude at FCz (congruent: t=5.66, p<0.001; incongruent: t=3.94, p<0.001), but not at Fz (congruent: t=-1.24, p=0.216; incongruent: t=1.19, p=0.237), reduced ERN amplitude at FCz (t=5.29, p<0.001), as well as reduced DSB (t=-2.92, p=0.004) and reduced Pe amplitude (t=-3.66, p<0.001) compared to the control group. Compared to the ADHD group, the preterm groups had significantly reduced MRT (t=-3.99, p<0.001), RTV (t=-4.41, p<0.001), DSF (t=2.36, p=0.020), N2 amplitude at Fz (congruent: t=1.99, p=0.049; incongruent: t=2.29, p=0.023) and FCz (congruent: t=2.86, p=0.005; incongruent: t=3.24, p=0.001). The preterm group did not differ from the ADHD group on DSB (t=-0.10, p=0.921), ERN (Fz: t=-1.32, p=0.190; FCz: t=1.07, p=0.285) and Pe amplitude (t=-0.27, p=0.791). The ADHD and control groups significantly differed on DSB, MRT, RTV, ERN amplitude at FCz and Pe amplitude (Michelini et al., 2016).

Among those born preterm, DIVA ADHD symptoms correlated with ERN amplitude at FCz (r=0.20, p=0.026), but not with ERN amplitude at Fz (r=0.12, p=0.199), Pe amplitude (r=-0.13, p=0.062), N2 amplitude at Fz (r=0.09, p=0.091) or N2 amplitude at FCz (r=-0.02, p=0.775).

**Appendix B**

*ERP results*

The random intercept model yielded a significant main effect of group for DSB (z=-3.17, p=0.002), N2 (z=5.24, p<0.001) and ERN (z=2.74, p=0.006) amplitude (Figures 1-4). No significant main effect of group emerged for DSF (z=1.29, p=0.197), MRT (z=0.40, p=686), RTV (z=0.98, p=329), errors (z=0.79, p=0.432) or Pe amplitude (z=-1.03, p=0.301).

Significant group-by-electrode site interactions emerged for N2 (z=5.02, p<0.001) and ERN (z=5.67, p<0.001). No significant group-by-congruency interaction emerged for N2 (z=-1.64, p=0.101), MRT (z=1.52, p=0.129), RTV (z=-0.24, p=0.813) or errors (z=-0.35, p=0.728). No significant group-by-congruency-by-electrode site interaction emerged for N2 amplitude (z=-0.79, p=0.428).

Post-hoc analyses revealed that the preterm group had significantly reduced N2 amplitude at FCz (t=4.78, p<0.001), but not at Fz (t=-0.60, p=0.551), and ERN amplitude at FCz (t=4.57, p<0.001), as well as reduced DSB (t=-2.92, p=0.004) and Pe amplitude (t=-2.46, p=0.015) compared to the control group. The preterm and control groups did not differ significantly on DSF (t=0.54, p=0.593). Compared to the ADHD group, the preterm groups had significantly reduced DSF (t=2.36, p=0.020), N2 amplitude at Fz (t=2.80, p=0.006) and FCz (t=3.64, p<0.001). The preterm group did not differ from the ADHD group on DSB (t=-0.10, p=0.921), ERN (Fz: t=-0.57, p=0.572; FCz: t=1.77, p=0.058) and Pe amplitude (t=-0.52, p=0.605). The ADHD and control groups significantly differed on DSB (t=-2.81, p=0.006), ERN amplitude at FCz (t=3.24, p=0.002) and Pe (t=-2.07, p=0.041) amplitude.

Among those born preterm, DIVA ADHD symptoms correlated with ERN amplitude at FCz (r=-0.19, p=0.01), but not with ERN amplitude at Fz (r=0.06, p=0.315), Pe amplitude (r=-0.14, p=0.081), N2 amplitude at Fz (r=0.11, p=0.053) or at FCz (r=-0.01, p=0.816).

**Appendix C**

The random intercept model yielded a significant main effect of group for MRT (z=-4.56, p<0.001), RTV (-3.08, p=0.002), DSB (z=-3.81, p<0.001), N2 (z=3.60, p<0.001), Pe (z=-3.10, p=0.002) and ERN (z=2.93, p=0.003) amplitude. No significant main effect of group emerged for DSF (z=0.67, p=0.500), or errors (z=0.97, p=0.332).

Significant group-by-electrode site interactions emerged for N2 (z=4.60, p<0.001) and ERN (z=5.43, p<0.001). No significant group-by-congruency interaction emerged for N2 (z=-1.68, p=0.093), MRT (z=0.88, p=0.380), RTV (z=0.40, p=0.688) or errors (z=1.65, p=0.098). A significant group-by-congruency-by-electrode site interaction emerged for N2 amplitude (z=2.67, p=0.008).

Post-hoc analyses revealed that the preterm group had significantly reduced N2 amplitude at FCz for both congruent (t=5.16, p<0.001) and incongruent (t=3.83, p<0.001) trials, reduced ERN at FCz (t=4.86, p<0.001), as well as reduced DSB (t=-4.18, p>0.001), MRT (t=-2.60, p=0.10) and Pe amplitude (-3.38, p=0.001) compared to the control group, with medium-to-large effect sizes. No significant differences between the preterm and control groups were found for RTV (t=-0.10, 0.921) and errors (t=1.01, 0.315). Compared to the ADHD group, the preterm groups had significantly decreased incongruent MRT (t=-4.50, p>0.001) and RTV (t=-4.31, p>0.001), as well as reduced N2 amplitude at FCz (t=3.00, p=0.003), medium-to-large effect sizes (Table 2 and Figure 4). The ADHD and control groups significantly differed on DSB (t=-4.37, p>0.001), incongruent MRT (t=3.37, p=0.001), RTV (t=4.98, p>0.001), ERN amplitude at FCz (t=4.21, p<0.001) and Pe (t=-4.32, p<0.001) amplitude, but not in terms of N2 amplitude (t=1.24, p=0.217). The preterm group did not differ from the ADHD group on ERN (t=1.09, p=0.277) and Pe (t=0.24, p=0.809) amplitude.

Among those born preterm, DIVA ADHD symptoms correlated with ERN amplitude at FCz (r=-0.17, p=0.041), but not with ERN amplitude at Fz (r=0.06, p=0.590), Pe amplitude (r=-0.09, p=0.227), N2 amplitude at Fz (r=0.07, p=0.350) or N2 amplitude at FCz (r=0.02, p=0.805).

**Appendix D**

|  | **ADHD** | **Preterm** | **Control** | **z-statistic** | **p-value** |
| --- | --- | --- | --- | --- | --- |
|  | n=32 | n=97 | n=59 | - | - |
| **GA in weeks (SD)** | 39.7 (1.4) | 33.4 (2.5) | 39.8 (1.3) | -17.5 | <0.001 |
| **GA range in weeks** | 37 - 42 | 26 - 36 | 37 - 43 | - | - |
| **IQ (SD)** | 96.5 (14.4) | 104.6 (12.7) | 107.9 (11.0) | -3.2 | 0.002 |
| **Age (SD)** | 16.5 (1.5) | 16.4 (1.0) | 16.5 (0.9) | -0.50 | 0.615 |
| **Age range** | 14.1-18.9 | 15.0-18.7 | 14.3-18.0 | - | - |
| **Males %** | 90.6 | 47.4 | 69.5 | 3.22 | <0.001 |
| **Conners’ parent rated ADHD symptom score (SD)** | 37.8 (11.1) | 10.9 (8.6) | 10.5 (7.2) | -1.30 | 0.302 |
| **BFIS score (SD)** | 15.6 (5.5) | 4.0 (6.0) | 4.1 (4.9) | -1.56 | 0.120 |

S1 Table1. Descriptive statistics.

*ERP results*

The random intercept model yielded a significant main effect of group for DSF (z=2.46, p=0.014), DSB (z=-2.44, p=0.015), N2 (z=6.05, p<0.001), Pe (z=-2.31, p=0.021) and ERN (z=2.91, p=0.004) amplitude. No significant main effect of group emerged for MRT (z=-0.22, p=0.824), RTV (z=-0.48, p=0.629) or errors (z=0.32, p=0.747).

Significant group-by-electrode site interactions emerged for N2 (z=2.03, p<0.043) and ERN (z=5.45, p<0.001) amplitude. A significant group-by-congruency interaction emerged for N2 (z=-2.67, p=0.007), but not for MRT (z=1.74, p=0.082), RTV (z=-0.37, p=0.710) or errors (z=-0.76, p=0.445). No significant group-by-congruency-by-electrode site interaction emerged for N2 amplitude (z=-0.79, p=0.428).

Post-hoc analyses revealed that the preterm group had significantly reduced N2 amplitude at Fz (congruent: t=2.69, p=0.008; but not incongruent: t=1.81, p=0.072) and FCz (congruent: t=5.81, p<0.001; incongruent: t=3.76, p<0.001) and ERN amplitude at FCz (t=4.22, p<0.001), as well as reduced DSB (t=-3.35, p=0.001) and Pe amplitude (t=-2.46, p=0.015) compared to the control group. The preterm and control groups did not differ significantly with regards to DSF (t=-0.13, p=0.897). Compared to the ADHD group, the preterm groups had significantly reduced N2 amplitude at Fz (congruent: t=2.81, p=0.006; incongruent: t=2.78, p=0.006) and FCz (congruent: t=3.34, p=0.001; incongruent: t=3.24, p=0.002), as well as significantly increased DSB (t=-4.20, p<0.001). The preterm group did not differ from the ADHD group on DSF (t=-0.66, p=0.510), ERN (Fz: t=-1.53, p=0.129; FCz: t=1.40, p=0.166) and Pe amplitude (t=-0.34, p=0.738). The ADHD and control groups significantly differed on DSB (t=-2.81, p=0.006), ERN amplitude at FCz (t=2.50, p=0.014) and Pe (t=-2.07, p=0.041) amplitude and incongruent N2 amplitude at FCz (t=2.15, p=0.034), but not on N2 at Fz (congruent: t=-0.69, p=0.493; incongruent: t=-1.28, p=0.205) or congruent N2 amplitude at FCz (t=0.77, p=0.433).

Among those born preterm, DIVA ADHD symptoms correlated with ERN amplitude at FCz (r=-0.16, p=0.042), but not with ERN amplitude at Fz (r=0.16, p=0.125), Pe amplitude (r=-0.14, p=0.06), N2 amplitude at Fz (r=0.06, p=0.429) or N2 amplitude at FCz (r=0.02, p=0.782).
